# Induction of p53-Dependent p21 Limits Proliferative Activity of Rat Hepatocytes in the Presence of Hepatocyte Growth Factor

**DOI:** 10.1371/journal.pone.0078346

**Published:** 2013-11-04

**Authors:** Yukiko Inoue, Tomoaki Tomiya, Takako Nishikawa, Natsuko Ohtomo, Yasushi Tanoue, Hitoshi Ikeda, Kazuhiko Koike

**Affiliations:** 1 Department of Gastroenterology, Graduate School of Medicine, The University of Tokyo, Tokyo, Japan; 2 Department of Clinical Laboratory Medicine, Graduate School of Medicine, The University of Tokyo, Tokyo, Japan; Osaka University Graduate School of Medicine, Japan

## Abstract

**Background:**

Hepatocyte growth factor (HGF), a potent mitogen for hepatocytes, enhances hepatocyte function without stimulating proliferation, depending on the physiological conditions. p53, a transcription factor, suppresses the cell proliferation by expressing p21^WAF1/CIP1^ in various tissues.

**Aim:**

To investigate the mechanism through which the hepatocytes maintain mitotically quiescent even in the presence of HGF.

**Methods:**

We studied the relationship between p53 and p21 expression and the effect of p53-p21 axis on hepatocyte proliferation in primary cultured rat hepatocytes stimulated by HGF. Hepatic p21 levels are determined serially after partial hepatectomy or sham operation in rats.

**Results:**

DNA synthesis was markedly increased by HGF addition in rat hepatocytes cultured at low density but not at high density. Cellular p53 levels increased in the hepatocytes cultured at both the densities. p21 levels were increased and correlated with cellular p53 levels in hepatocytes cultured at high density but not at low density. When the activity of p53 was suppressed by a chemical inhibitor for p53, cellular p21 levels were reduced, and DNA synthesis was increased. Similarly, p21 antisense oligonucleotide increased the DNA synthesis. In rats after partial hepatectomy, transient elevation of hepatic p21 levels was observed. In contrast, in sham-operated rats, hepatic p21 levels were increased on sustained time scales.

**Conclusion:**

p53-related induction of p21 may suppress hepatocyte proliferation in the presence of HGF in the setting that mitogenic activity of HGF is not elicitable.

## Introduction

Proliferation of hepatocytes occurs following the loss of parenchymal cells, while hepatocytes are usually mitotically quiescent. Hepatocyte growth factor (HGF), originally identified as a potent mitogen for hepatocytes in culture, has a pluripotent effect on various types of cells [1]–[6]. Previous reports indicate that circulating HGF levels in humans are increased with various degrees in physiological and pathological conditions such as acute hepatitis, fulminant hepatic failure, chronic hepatitis, liver cirrhosis, renal failure, post-partial hepatectomy and post-non-hepatectomized abdominal surgery [3], [4], [7]–[10]. In the liver of experimental animal models, mitogenic, anti-inflammatory, anti-apoptotic and anti-fibrogenetic activities of HGF have been observed [3], [7]. In primary cultured hepatocytes, HGF addition has been shown to facilitate proliferation or function of the cells depending on the culture condition [2], [11]. The mechanism is still under investigation if the specific activities of HGF are selectively expressed. Previously, we reported that HGF exerted mitogenic activity on hepatocytes through the induction of p53, a transcription factor, which increased production of transforming growth factor α (TGF-α), a complete mitogen for hepatocytes [12]–[14]. However, the mechanism is unknown through which hepatocytes maintain mitotically quiescent when HGF exerts other activities.

Though recent several reports including ours indicate that p53 can stimulate cell proliferation by the specific induction of promoters for growth-associated factors such as TGF-α, p53 is generally recognized as a ‘tumor suppressor gene’, because, in some pathophysiological conditions, it up-regulates p21, which arrests cell cycle at G1 phase, and inhibits cell proliferation both in vitro and in vivo [14]–[17]. While p21 expression can be induced by growth-inhibitory stimuli such as DNA damage [18]–[22], recent reports indicate the possibility that addition of some growth factors induces p21, and suppresses DNA synthesis especially in malignant cell lines[18], [23]–[28]. However, relationship between p53 and p21 and its significance in non-malignant cells including hepatocytes in the presence of growth factors is still under investigation.

In this paper, we showed that p21 was up-regulated by HGF stimulation through the induction of p53, and suppressed hepatocyte proliferation in the setting that mitogenic activity was not elicitable.

## Materials and Methods

### Assay for p53 of cultured hepatocytes

Hepatocyte extracts were prepared according to the protocol from the manufacturer of the p53 enzyme-linked immunosorbent assay (ELISA) kit (Roche Molecular Biochemicals, Germany) [14].

### Preparation of liver and cultured hepatocytes for p21 assay

Liver tissues and cultured hepatocytes were homogenized in the low salt resuspension buffer (pH 7.4, 50 mmol/L tris (hydroxymethyl) aminomethane, 5 mmol/L ethylenediaminetetraacetic acid, 0.2 mmol/L phenylmethylsulfonyl fluoride 1 µg/mL pepstatin and 0.5 µg/mL leupeptin). The suspensions were incubated with p21 antigen extraction agent (1.0 M potassium chloride, 6% zwittergent (Calbiochem, CA)), and centrifuged. The resultant supernatants were applied to ELISA described below.

### Assay for p21 in liver extracts and cultured hepatocytes

The sandwich ELISA for p21 was developed using polyclonal anti p21 IgG (Santa Cruz, CA) and monoclonal p21 antibody (Santa Cruz, CA) as capture and detector antibodies, respectively. Horseradish peroxidase conjugated goat anti-mouse IgG (Zymed, CA) was used to detect the antibody-p21 complex.

The standard curve for p21 (1–164, full length amino acids, Santa Cruz Biotechnology, CA) of this assay made with the buffer showed the lower limit at 1.25 ng/mL. When the sample of rat liver prepared for p21 assay was diluted with the buffer, the dilution curve was similar to the standard curve. When p21 protein was diluted with sample of rat liver or hepatocytes prepared for p21 assay, the dilution curve was similar to the standard curve.

### Determination of 5-bromo-2′-deoxy-uridine (BrdU) incorporation and total protein content of cultured hepatocytes

Incorporated BrdU was determined by ELISA, using BrdU labeling and the detection kit III (Roche Molecular Biochemicals, Germany). The total cellular protein was measured by Bradford's method [29].

### Experiments with cultured hepatocytes

Hepatocytes were isolated from rat livers according to Seglen's method [30]. The isolated cells were cultured at densities of either 1.2×10^5^ cells/cm^2^ (high density culture) or 2.5×10^4^ cells/cm^2^ (low density culture) in the medium and incubated for 27 hours as we previously reported [14]. The medium was changed to Williams' medium E (WE) containing 10% fetal calf serum (FCS), various concentrations of HGF and 0.1 mmol/L BrdU. The cells were harvested serially for the determination of both the cellular p53 and p21 levels and, the BrdU incorporation into cellular DNA.

To study the effect of inhibition of p53 function on p21 levels and DNA synthesis in hepatocytes, the hepatocytes were cultured in WE containing 10% FCS, with or without 10 ng/mL HGF, 1 mmol/L BrdU and various concentrations of pifithrin-α (Alexis Biochemicals, CA) dissolved in dimethyl sulfoxide (DMSO) or the same concentrations of DMSO [31]. The cells were harvested 18 hours later to determine the cellular p21 levels and 24 hours later to determine the BrdU incorporation into cellular DNA.

To examine the effect of inhibition of p21 production on hepatocyte DNA synthesis, the hepatocytes were cultured in WE containing 10% FCS, 10 ng/mL HGF, 1 mmol/L BrdU and various concentrations of either p21 antisense oligonucleotide (5′-GACATCACCAGGATCGGACAT-3′), complementary to position 85–105 of rat p21 mRNA, or nonsense oligonucleotide (5′-GCAACGCTACTACGCAAGTAG-3′), containing the same numbers of G, C, A, and T as the p21 antisense oligonucleotide [32]. The cells were harvested as above, to determine the cellular p21 levels and the BrdU incorporation into cellular DNA.

### Animal experiments

Five to six-weeks-old Male Sprague-Dawley rats (Japan SLC, Japan) were subjected to either of two-thirds partial hepatectomy (PH) or sham operation under diethyl ether anesthesia. In sham-operated rats, the abdomen was cut open under similar anesthesia, and the liver was briefly exposed outside the peritoneal cavity. The rats were serially anesthetized with diethyl ether. The liver was perfused through the portal vein with saline. After a near total exsanguination, the liver was excised and used for the p21 assays.

All animal study protocols conformed to and approved by the guideline of the Faculty of Medicine, University of Tokyo for humane care.

### Statistical analyses

The differences between two unpaired samples were defined as significant when the p-values by both the Student's *t*-test and the Mann-Whitney *U* test were less than 0.05. The dose related effects were tested by one-way analysis of variance followed by Spearman's correlation test.

## Results

### Changes in DNA synthesis of cultured rat hepatocytes after HGF treatment

**Figure 1:**
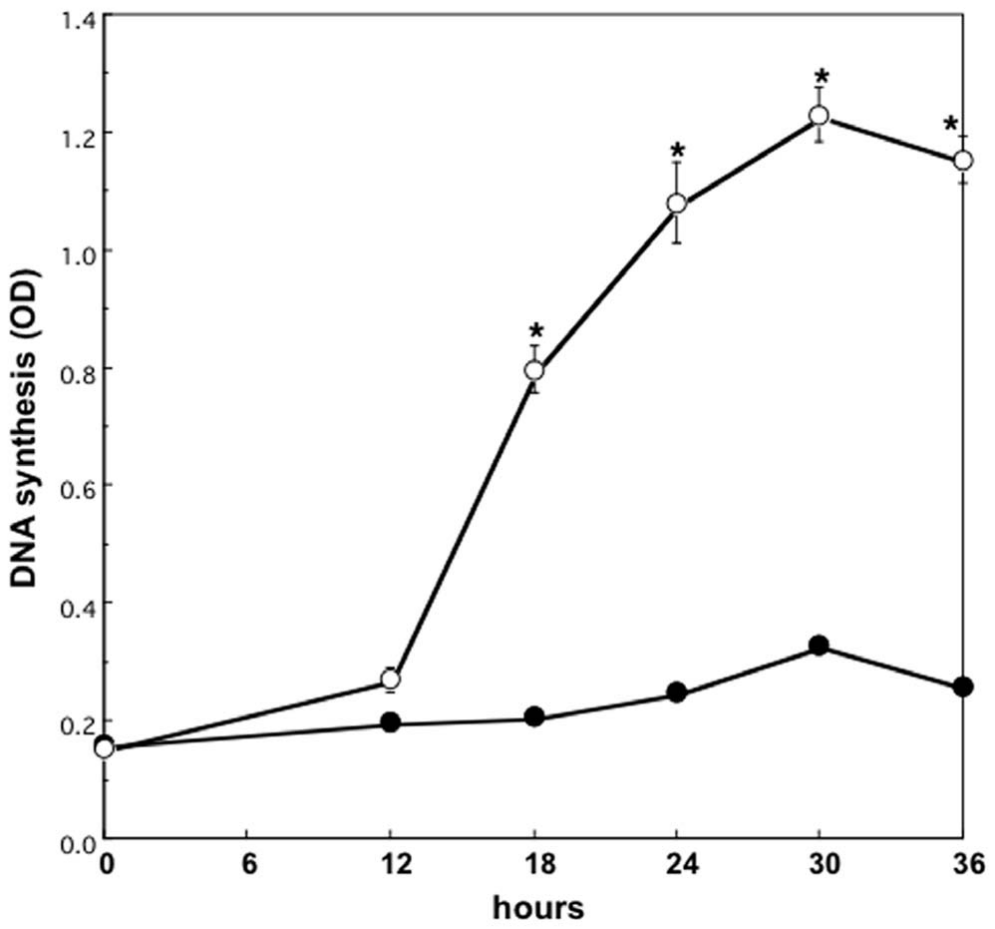
Changes in DNA synthesis of hepatocytes after HGF treatment. Rat hepatocytes were cultured in WE containing 10% FCS, 10 ng/mL HGF and 0.1 mmol/L BrdU, and were harvested serially. Closed circles denote hepatocytes cultured at high density. Open circles denote hepatocytes cultured at low density. Data are mean ± SEM of eight dishes. *p<0.01 compared with the values cultured for 0 hours.

We determined the effect of cell density on DNA synthesis of cultured hepatocytes simulated by HGF. The addition of 10 ng/mL HGF to the medium caused only minor increase on DNA synthesis in hepatocytes cultured at high density. In hepatocytes cultured at low density, DNA synthesis increased after 12 hours of incubation, peaked at 24–30 hours and decreased thereafter by HGF addition (Figure 1). DNA synthesis was not induced significantly in high density cultured hepatocytes by HGF treatment.

### Cellular p53 and p21 levels in cultured rat hepatocytes treated with HGF

To investigate the effect of HGF on p53 and p21 expressions and their relationship in proliferating and non-proliferating rat hepatocytes, we determined p53 and p21 protein levels in cultured hepatocytes at low and high density in the presence of various concentration of HGF.

**Figure 2:**
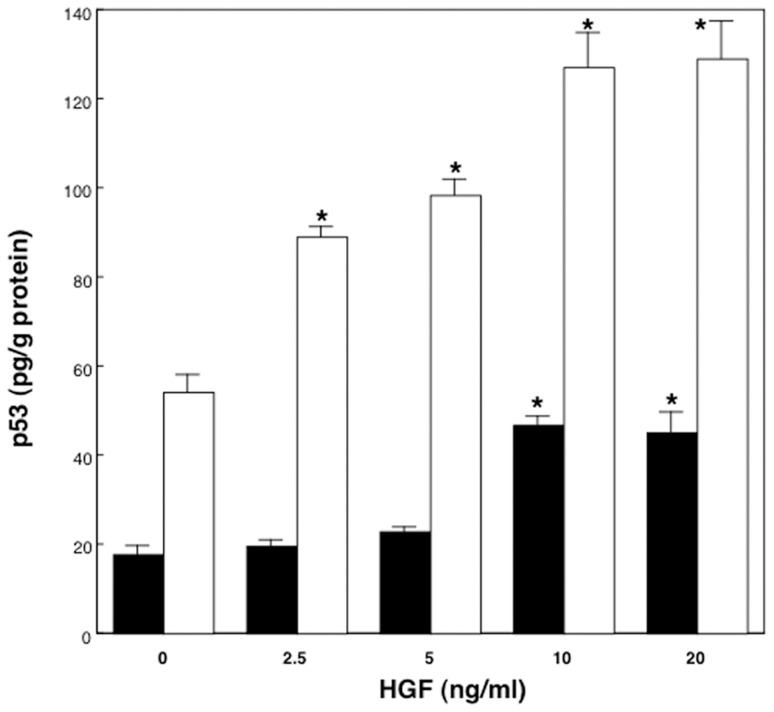
Cellular p53 levels in hepatocytes treated with HGF. Rat hepatocytes were cultured in WE containing 10% FCS and various concentration of HGF for 18 hours. Closed bars denote hepatocytes cultured at high density. Open bars denote hepatocytes cultured at low density. Data are mean ± SEM of four dishes. *p<0.01 compared with the values in the absence of HGF.

As shown in Figure 2, when rat hepatocytes were cultured at high density with HGF, p53 levels increased at 10 ng/mL or 20 ng/mL of HGF addition (F = 32.5, p<0.01; r = 0.84, p<0.01). In hepatocytes cultured at low density, HGF addition also increased cellular p53 levels significantly in a dose-related manner up to 10 ng/mL of HGF (F = 23.4, p<0.01; r = 0.90, p<0.01) (Figure 2).

The levels of p53 in high density cultured hepatocytes treated with 10 ng/mL HGF increased after 6 hours and reached a maximum at 24 hours (data not shown), while, in low density cultured hepatocytes treated with 10 ng/mL HGF, p53 levels significantly increased from 6 hours and peaked at 18 to 24 hours of incubation, similar to our previous report [14].

**Figure 3:**
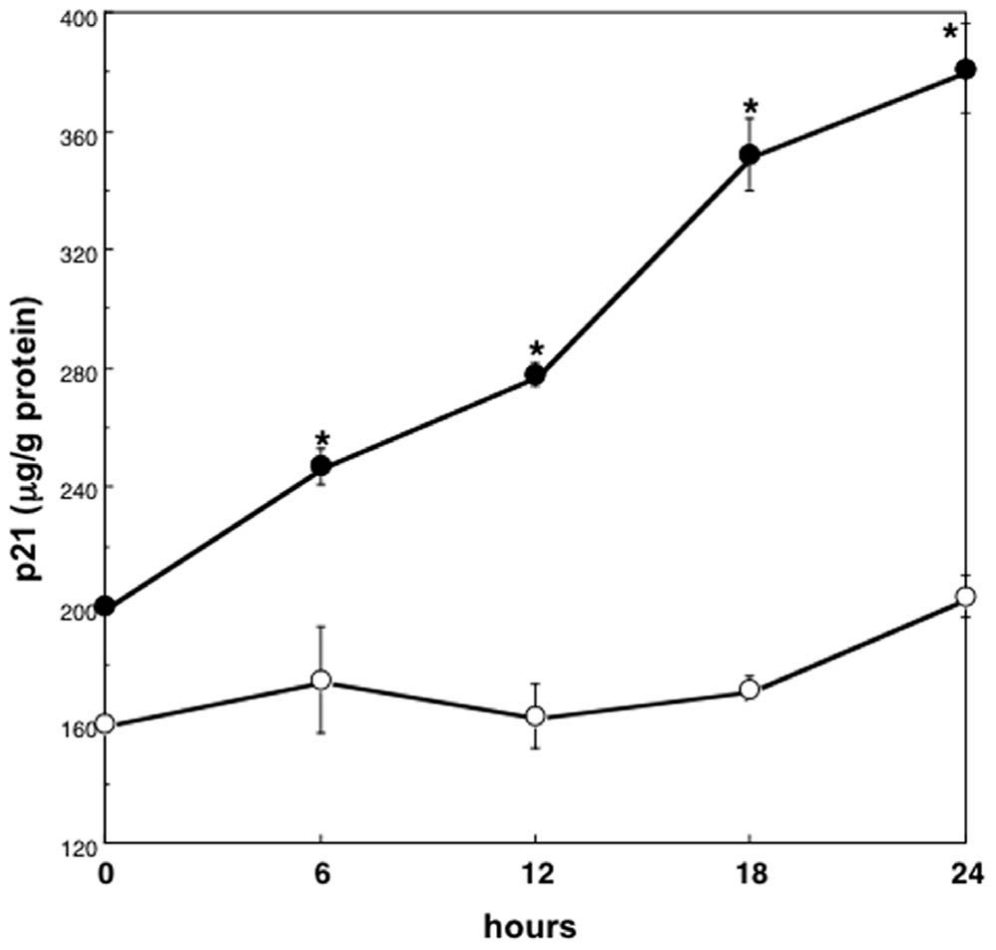
Serial changes in p21 protein levels of hepatocytes treated with HGF. Rat hepatocytes were cultured at high density in WE containing 10% FCS and 10 ng/mL HGF, and were harvested serially. Closed circles denote hepatocytes cultured at high density. Open circles denote hepatocytes cultured at low density. Data are mean ± SEM of four dishes. *p<0.01 compared with the values cultured for 0 hours.

**Figure 4:**
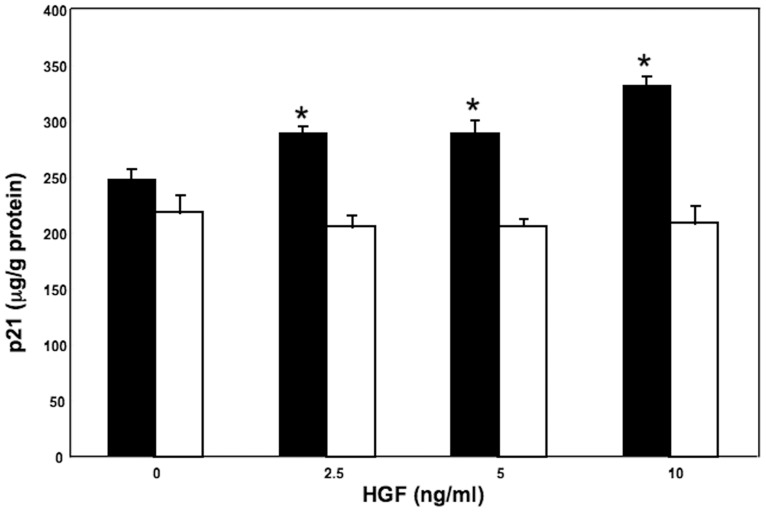
Cellular p21 levels in hepatocytes treated with HGF. Rat hepatocytes were cultured in WE containing 10% FCS and various concentration of HGF for 18 hours. Closed bars denote hepatocytes cultured at high density. Open bars denote hepatocytes cultured at low density. Data are mean + SEM of four dishes. *p<0.05 compared with the values in the absence of HGF.

**Figure 5:**
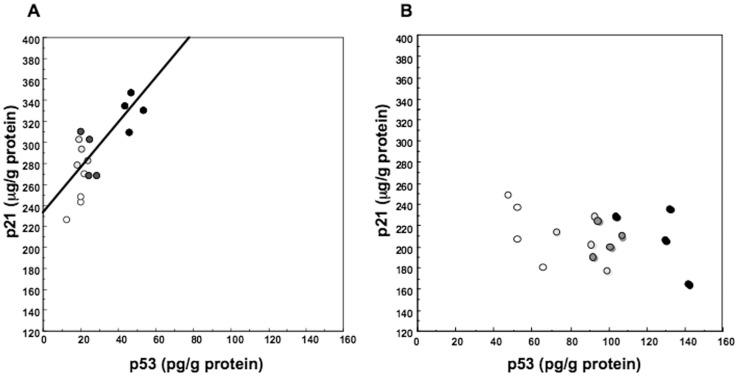
Cellular p53 and p21 levels in hepatocytes treated with HGF. Rat hepatocytes were cultured in WE containing 10% FCS and various concentrations of HGF for 18 hours. Open circles denote hepatocytes cultured in the absence of HGF. Dotted open circles denote hepatocytes cultured with 2.5 ng/mL HGF. Dotted closed circles denote hepatocytes cultured with 5 ng/mL HGF. Closed circles denote hepatocytes cultured with 10 ng/mL HGF. (A) Hepatocytes cultured at high density. (B) Hepatocytes cultured at low density.

The levels of p21 protein in high density cultured hepatocytes treated with 10 ng/mL HGF increased in a time dependent manner (Figure 3). When hepatocytes cultured at high density were treated with HGF at increasing concentrations, p21 levels at 18 hours after HGF addition increased in a dose-related manner (F = 73.0, p<0.01; r = 0.88, p<0.01), and correlated with the cellular p53 levels (r = 0.69; p<0.01) (Figure 4 and 5A). p21 levels in low density cultured hepatocytes were not increased by HGF addition, nor correlated with p53 levels (Figure 3, 4 and 5B).

p21 levels were increased in a dose related manner by HGF and correlated with p53 levels at high density cultured hepatocytes, while, at low density cultured hepatocytes, p53, but not p21, levels were increased by HGF and there was no correlation between p53 and p21 levels.

### The effect of pifithrin-α on p21 levels and BrdU incorporation in rat hepatocytes cultured at high and low density in the presence or absence of HGF

To elucidate the relationship between p53 expression and p21 expression as well as DNA synthesis in hepatocytes at non-proliferating condition even in the presence of HGF, we determined the effect of pifithrin-α, a chemical inhibitor of p53, on p21 levels and BrdU incorporation of hepatocytes cultured at high density in the presence of HGF.

**Figure 6:**
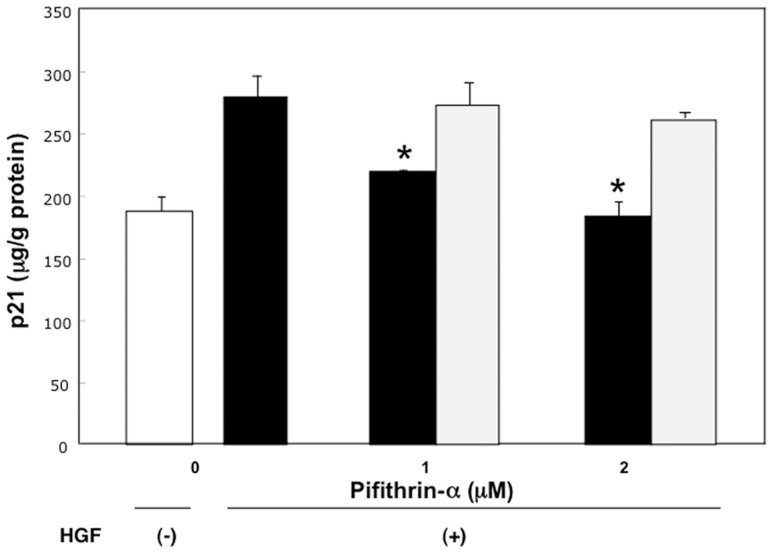
The effect of pifithrin-α on p21 levels of hepatocytes in the presence of HGF. Rat hepatocytes were cultured at high density in WE containing 10% FCS, 10 ng/mL HGF, along with various concentrations of pifithrin-α, the chemical inhibitor of p53, dissolved in DMSO, or DMSO of the same concentration for 18 hours. An open bar denotes hepatocytes cultured in the absence of HGF. Closed bars denote hepatocytes cultured with pifithrin-α in the presence of 10 ng/mL HGF. Dotted bars denote hepatocytes cultured with DMSO in the presence of 10 ng/mL HGF. Data are mean ± SEM of four dishes. *p<0.05 compared with the values treated only with HGF or values treated with HGF and DMSO.

The levels of p21 treated with 10 ng/mL HGF for 18 hours in hepatocytes at high density were reduced by the addition of pifithrin-α, a chemical inhibitor of p53, when compared with the addition of vehicles (p<0.01) (Figure 6).

In contrast, the levels of p21 in hepatocytes at high density treated without HGF for 18 hours, the values of p21 protein were 211.5±24.9 µg/g protein (mean±standard error), and did not show any significant changes by the addition of pifithrin-α when compared with the addition of vehicles (202.2±57.4 µg/g protein). In addition, the levels of p21 in hepatocytes cultured at low density treated with 10 ng/mL HGF for 18 hours were not affected by pifithrin-α treatment (121.7±18.1 µg/g protein vs137.6±21.8 µg/g protein).

**Figure 7:**
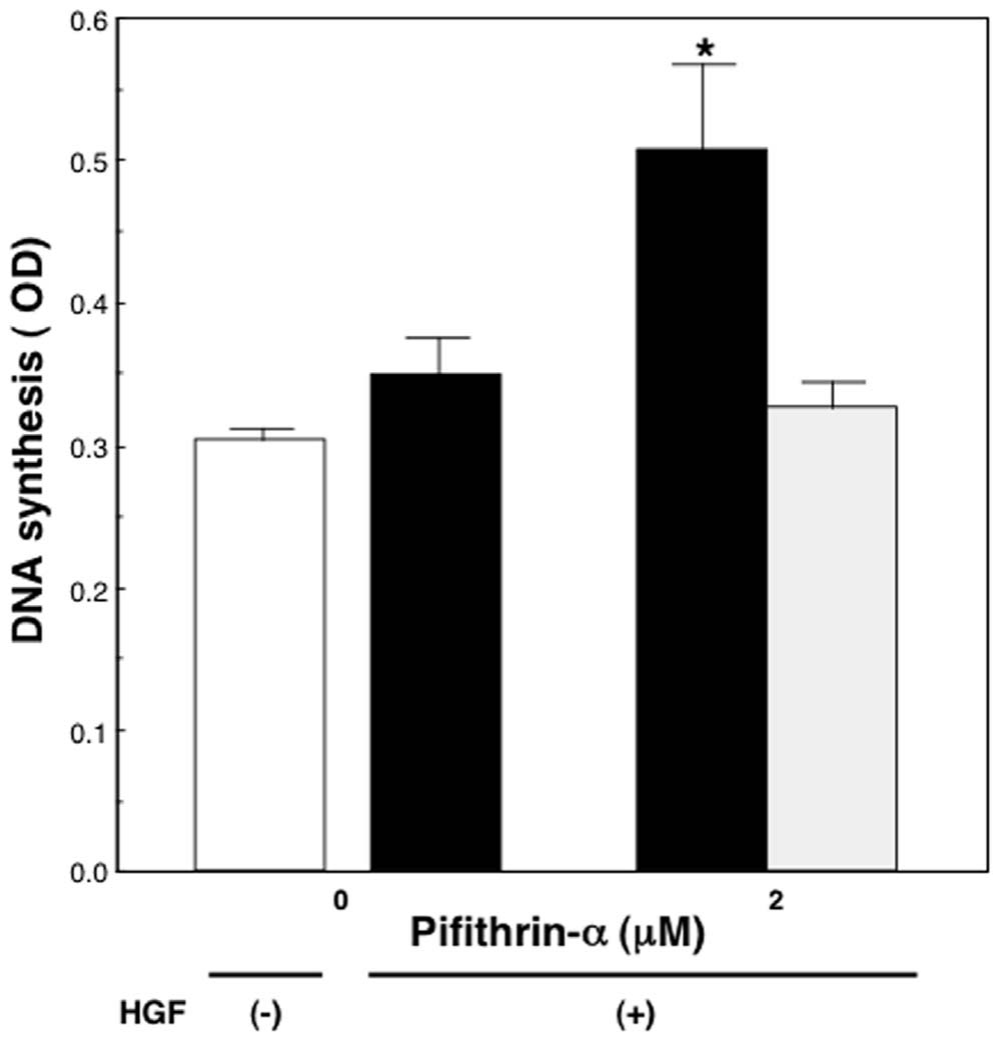
The effect of pifithrin-α on DNA synthesis of hepatocytes in the presence of HGF. Rat hepatocytes were cultured at high density in the same medium as described in the legend of Figure 6, except that the medium contained 1 mmol/L BrdU, for 24 hours. An open bar denotes hepatocytes cultured in the absence of HGF. Closed bars denote hepatocytes cultured with pifithrin-α in the presence of 10 ng/mL HGF. A dotted bar denotes hepatocytes cultured with DMSO in the presence of 10 ng/mL HGF. Data are mean ± SEM of eight dishes. *p<0.01 compared with the values treated only with HGF or values treated with HGF and DMSO.

The levels of BrdU incorporation treated with 10 ng/mL of HGF for 24 hours were increased significantly by the addition of 2 µM of pifithrin-α, when compared with the addition of vehicles (p<0.01) (Figure 7). The total cellular protein levels were not affected by the addition of either pifithrin-α or vehicles (data not shown).

p21 levels were reduced and DNA synthesis was significantly increased by pifithrin-α a chemical inhibitor of p53, in the presence of HGF in high density cultured hepatocytes.

### The effect of p21 antisense oligonucleotides on BrdU incorporation in rat hepatocytes cultured at high density in the presence of HGF

**Figure 8:**
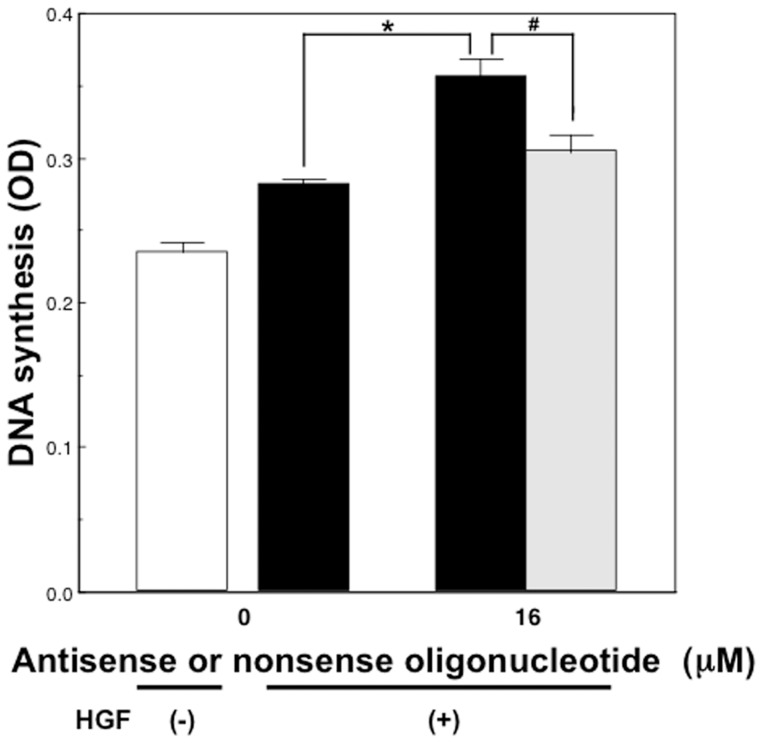
The effect of p21 antisense on DNA synthesis of hepatocytes in the presence of HGF. Rat hepatocytes were cultured at high density in WE containing 10% FCS, 10 ng/mL HGF, 1 mmol/L BrdU, along with various concentrations of either p21 antisense or nonsense oligonucleotide for 24 hours. An open bar denotes hepatocytes cultured in the absence of HGF. Closed bars denote hepatocytes cultured with p21 antisense oligonucleotide in the presence of 10 ng/mL HGF. A dotted bar denotes hepatocytes cultured with nonsense oligonucleotide in the presence of 10 ng/mL HGF. Data are mean ± SEM of eight dishes. *p<0.05 compared with the values treated only with HGF, and #p<0.01 compared with the values treated with HGF and nonsense oligonucleotide.

We investigated the effect of suppression of p21 expression on DNA synthesis in non-proliferationg hepatocytes in the presence of HGF. BrdU incorporation in hepatocytes cultured at high density in the presence of 10 ng/mL of HGF was significantly increased after a 24-hour exposure to p21 antisense oligonucleotide, when compared with that of hepatocytes treated with the nonsense oligonucleotide (p<0.01) (Figure 8). The total cellular protein levels were not affected by the addition of either oligonucleotide (data not shown). Suppression of p21 expression increased the DNA synthesis in the presence of HGF in high density cultured hepatocytes.

### p21 levels in the liver after two-thirds PH or sham operation in rats

**Figure 9:**
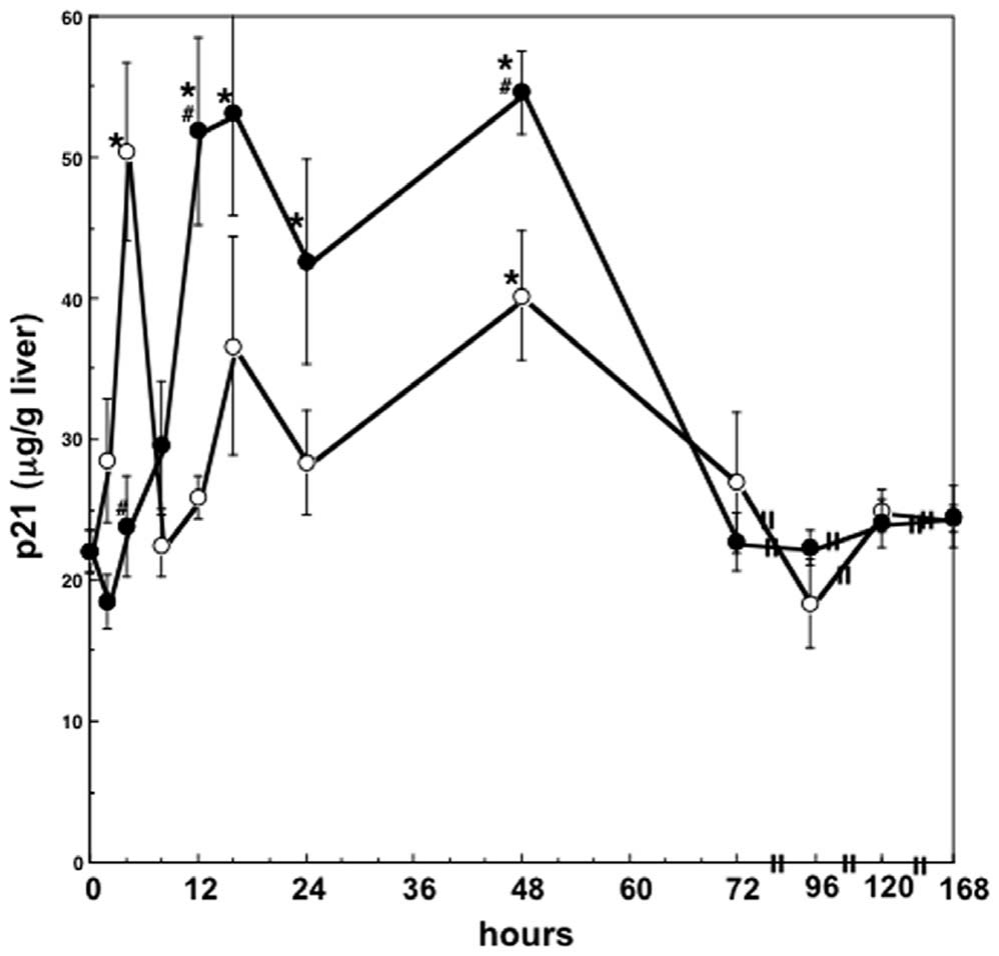
Changes in hepatic p21 levels after two thirds partial hepatectomy in rats. Data are mean ± SEM of four rats. Open and closed circles indicate the hepatic p21 levels in partially hepatectomized rats and in sham-operated rats, respectively. *p<0.05 compared with the values at 0 hour, and #p<0.05 compared with the values of partially hepatectomized rats.

To study p21 expression profile in regenerating and quiescent rat liver, we determined hepatic p21 levels in rats after PH and sham operation. Hepatic p21 levels were increased to the maximal levels at 4 hours, and decreased to the basal levels at 8 hours after PH with minor increase at 48 hours. In contrast, hepatic p21 levels of sham-operated rats were increased up to 2.5-fold higher than preoperative levels at 12 to 48 hours and decreased to the preoperative level at 72 hours. Hepatic p21 levels were significantly higher in sham-operated rats than in rats after PH except within 4 hours after surgery (Figure 9). In sham-operated rats, hepatic p21 levels were increased on sustained time scales while only transient elevation was observed in partial hepatectomized rats.

## Discussion

We confirmed that DNA synthesis was not induced significantly in hepatocytes cultured at high density even in the presence of HGF, as we previously reported [2]. HGF seems to increase p53 and p21, and maintain mitotically quiescent in the hepatocytes.

We determined p21 protein levels *in vitro* and *in vivo* using newly developed ELISA system. Although p21 expression has been extensively studied in proliferating and non-proliferating hepatocytes and in the liver with or without regenerative stimuli [33]–[36], the results are inconsistent. In most of these reports, p21 mRNA levels were studied, or p21 protein levels were determined by Western blotting. When p21 synthesis is induced, p21 mRNA levels are generally up-regulated. Furthermore, p21 synthesis is further increased at the post-transcriptional level in hepatocytes [37]. Thus, in this study, we determined p21 protein expressions which may function as transcription factor. In addition, to avoid the unstable results, we determined p21 protein levels utilizing a quantitative method which was sensitive enough to detect p21 during observation periods *in vitro* and *in vivo*.

We suppressed p53 activity and p21 expression using pifithrin-α and a p21 antisense oligonucleotide, respectively. Pifithrin-α disturbs the nuclear transport of p53 leading to the inhibition of the function of p53 *in vitro* and *in vivo* [31], [38]. We used pifithrin-α at the same concentration as previously reported in hepatocytes [14], [38]. The efficacy of the p21 antisense oligonucleotide was also previously reported in other cell culture systems [32].

Previously, we reported that the addition of HGF to the medium increased p53 contents in hepatocytes cultured at low density followed by the increase of DNA synthesis by hepatocytes [14]. In this present paper, we showed that the p53 levels were also increased by HGF treatment in hepatocytes cultured at high density which did not show apparent burst of DNA synthesis. The mechanisms responsible for the increase of p53 in hepatocytes by HGF are still undefined. However, it has been reported that activation of mitogen activated protein (MAP) kinase influences transcription factors including p53 [39]. Considering that MAP kinase is thought to mediate the intracellular effects of HGF [40], HGF might increase p53 through the interaction of MAP kinase and p53. Recently, the relationship between growth factors and p53 has been shown in a couple of reports. HGF was shown to increase p53 expression in a rat epithelial cell line, and insulin-like growth factor I was reported to induce p53 expression in cardiac muscle cells [41], [42]. In primary cultured rat hepatocytes, epidermal growth factor (EGF) was shown to induce p53 expression in a phosphatidylinositol-3 kinase-dependent way [36]. In addition, p53 null hepatocytes were reported to be refractory to the stimulation of EGF [43].

p21 is known to be induced by p53-dependent and -independent mechanisms according to the cell types and situations [18]. Following DNA damage, p53 appears to be necessary for p21 induction in various kinds of cell types [18]. Many experiments showed that p21 was the major effector of p53 in inducing growth arrest in malignant cells [17], [18]. Furthermore, p21 was reported to be induced by p53 and negatively regulate cell proliferation in normal fibroblasts without DNA damage [44]. In addition, expression of the p53-induced p21 was greatly diminished by targeting p53 with anti-p53 antibody, and the cells reentered S-phase in fibroblasts [45]. We showed that cellular levels of p21 correlated with those of p53 and suppression of p53 activity by pifithrin-α resulted in the decrease of p21 levels followed by an increase of DNA synthesis in hepatocytes cultured at high density. p21 seems to be induced by p53 dependent mechanism in the present culture system of hepatocytes leading to suppression of proliferation.

Previous several reports have shown that growth factors can induce p21 production and suppress cell proliferation. Transient induction of p21 mRNA following stimulation of growth factors such as EGF, platelet-derived growth factor and fibroblast growth factor is reported in several cell lines, leading to cell cycle arrest [18], [26]–[28], [46]. p21 was up-regulated by HGF addition, and mediated growth inhibition in a hepatoma cell-line [23]–[25]. In primary cultured rat and mouse hepatocytes, p21 is reported to be induced by EGF and to have a role in the blockage of hepatocyte replication of the second round but not of the first round [33]. In addition, Wierod et.al reported that EGF induced p21 through the activation of p53 in primary cultured rat hepatocytes [36]. However, they noted that EGF-induced p21 might positively regulate DNA synthesis, since stimulatory effect of EGF on DNA synthesis was abrogated when p53 was inhibited, and rescued by ectopical p21 addition [36]. It was reported that the role of p21 might differ by its concentrations [47]. To examine the role of intrinsic p21 in regulating DNA synthesis of hepatocytes, the effect of p21 inhibition in the presence of EGF should be studied. In our culture system using primary cultured rat hepatocytes and HGF, we showed that suppression of HGF-induced increase of p21 production positively regulated DNA synthesis by hepatocytes cultured at high density, suggesting that up-regulation of p21 maintained mitotically quiescent in hepatocytes in the presence of HGF. No apparent change of p21 expression was caused by pifithrin-α in hepatocytes cultured without HGF treatment, suggesting the effect of p53 inhibition on baseline p21 seemed to be minimal. In the high density cultured hepatocytes, protein production such as albumin is increased by HGF addition [2], [11]. In contrast, in hepatocytes cultured at low density, we previously reported that HGF induced TGF-α production through the induction of p53, and hepatocyte proliferation occurred [14]. It is possible to speculate that induction of diverse effector genes of p53 plays a role in the expression of different activities of HGF. Further investigations would be required to clarify the mechanism(s) of the selective expression of p53 related genes in hepatocytes in different conditions stimulated by HGF.

Previous experiments in rats showed that hepatic levels of p21 protein after PH determined by Western blot analysis were increased at two time points, first immediately after resection and second after the peak of DNA synthesis. In sham operated rats, the p21 levels were almost undetectable throughout the 7-day time course [34]. Controversially, some reports demonstrated that hepatic p21 protein levels determined by Western blot analysis were decreased in partially hepatectomized rats and increased in sham-operated rats. The maximum decrease was observed immediately before the peak DNA synthesis, with increased p53 levels after PH, while the levels began to increase immediately after sham operation, and continued up to 48 hours of observation [48]. In mice after PH, p21 expression was reported to be increased before DNA synthesis of hepatocytes and reached maximum after the peak of hepatocyte DNA synthesis on Western blot analysis [49]. In the present study, hepatic p21 protein levels were increased immediately after PH, but returned to preoperative levels within 8 hours, while a sustained increase of p21 levels was observed after sham operation up to 48 hours. The reason for the discrepancy of the data is unclear. The difference of the assay system might influence the observations. Previously, we reported that hepatic p53 levels as well as hepatic and circulating HGF levels were increased after sham operation without the increase of hepatocyte proliferation [12], [14], while, in rats after PH, hepatic p53 levels were increased and reached maximal levels, when hepatic HGF levels have been shown to reach maximum prior to an increase in hepatocyte proliferation [13], [14]. These observations raise the possibility that the increase of p21 is related to keep quiescent when hepatocyte proliferation is not physiologically required even though HGF levels are increased. Previous studies described that c-Jun controlled hepatocyte proliferation by a p53/p21-dependent mechanism in mice [50]. p21 knock out mice demonstrated markedly accelerated hepatocyte proliferation compared to cogenic wild-type mice after PH [49]. These reports support our hypothesis. Significance of the transient increase of hepatic p21 levels after PH is still to be elucidated. The role of p21 may change depending on its concentration; p21 promotes cyclin/cdk complex assembly at low concentration, whereas, at higher concentrations, p21 is inhibitory to cdk in human normal fibroblast and human osteosarcoma cell [47]. The levels and the timing of the increase might influence the activity of p21.
